# Sex-Dependent Effects of Prenatal Stress on Seizure Susceptibility and Neurodegeneration in Neonatal Rats

**DOI:** 10.3390/brainsci15111220

**Published:** 2025-11-13

**Authors:** Daniel Antonio Cruz-Rojas, Luis Beltrán-Parrazal, Consuelo Morgado-Valle, Grecia Herrera-Meza, Aleph A. Corona-Morales, Joel Martínez-Quiroz, Brenda Martínez-Rojas, María-Leonor López-Meraz

**Affiliations:** 1 Doctorado de Investigaciones Cerebrales, Universidad Veracruzana, Xalapa 91190, Mexico; zs18024965@estudiantes.uv.mx (D.A.C.-R.); zS21023644@estudiantes.uv.mx (B.M.-R.); 2 Instituto de Investigaciones Cerebrales, Universidad Veracruzana, Xalapa 91190, Mexico; lubeltran@uv.mx (L.B.-P.); comorgado@uv.mx (C.M.-V.); 3 Unidad de Estudios de Posgrado, Benemérita Escuela Normal Veracruzana, Xalapa 91017, Mexico; greehem@gmail.com; 4 Facultad de Nutrición, Universidad Veracruzana, Xalapa 91017, Mexico; alecorona@uv.mx; 5 Facultad de Química Farmacéutica Biológica, Universidad Veracruzana, Xalapa 91000, Mexico; joemartinez@uv.mx

**Keywords:** *status epilepticus*, maternal stress, neonates, neurodegeneration, sex

## Abstract

Background: Prenatal stress affects fetal neurodevelopment and may increase the risk of seizures. This study aimed to analyze the impact of maternal restraint stress during pregnancy on neonatal *status epilepticus* (SE) in rats. Methods: Pregnant Wistar rats were subjected to restraint stress from gestation days 12 to 20. Offspring were assessed for body weight, size, and corticosterone levels. SE was induced in postnatal day 7 rats using the lithium–pilocarpine model. Neurodegeneration was analyzed using Fluoro-Jade C staining. Results: Maternal restraint stress resulted in reduced weight gain for the mothers and lower body weight and size for their offspring. Stressed neonates exhibited higher levels of serum corticosterone. Male neonates exhibited shorter latency to stage 1 seizures and increased hippocampal neurodegeneration compared with control males, whereas female neonates were largely unaffected. Conclusions: Maternal restraint stress produced only mild, sex-dependent effects on neonatal seizure susceptibility, affecting males but not females, suggesting a limited yet selective influence of prenatal stress on early brain vulnerability.

## 1. Introduction

Maternal stress and anxiety are common issues experienced throughout pregnancy [1]. Research has shown that maternal stress acts as a prenatal programming factor that influences fetal and child neurodevelopment, leading to complications such as premature births, low birth weight, and neurological disorders [2,3,4].

The impact of prenatal stress on neurological development has been investigated by the scientific community from a preclinical perspective [5,6]. Specifically, studies have explored how prenatal stress, induced by treatments like betamethasone, acute immobilization, or exposure to a predator, affects seizure susceptibility. In these studies, the generation of seizures triggered by N-methyl-D-aspartate (NMDA) in postnatal day (P) 15 rat pups was analyzed as a model for cryptogenic infantile spasms. Researchers discovered that stressed animals experienced spasms more quickly and frequently than control rats and with shorter latencies [7,8,9,10]. Another study reported that restraint stress during gestational days 15–17 did not influence the latency or duration of pentylenetetrazol-induced tonic–clonic seizures in P14 or P21 rats; however, P14 rats experienced a greater number of seizures compared to the control group [11]. Edwards et al. [12] utilized electrical hippocampal kindling as their experimental seizure model in infant P 14 rats. They used repeated maternal restraint under bright light during gestation as the prenatal stress model. This prenatal stress, particularly during mid/late gestation, increased seizure susceptibility by lowering the after-discharge threshold and accelerating kindling development.

Regarding *status epilepticus* (SE), a prevalent prolonged seizure condition in neonates and infants that causes neuronal injury [13,14], evidence indicates that prenatal stress, from physical restraint or exposure to a predator during gestation days 15–17, increased mortality rates following pilocarpine-induced seizures in P25 rats [15]. However, the effects of maternal restraint during the first, second, or third weeks of gestation, or throughout the entire gestational period, varied on pilocarpine-induced seizures in P18–19 rats. Stress during late gestation and throughout the entire gestation period, but not during early gestation, enhanced seizure severity (characterized by increased duration, higher frequency, and reduced latency) and mortality after pilocarpine administration [16]. Conversely, prenatal exposure to psychological stress (i.e., predator stress) did not affect the severity of febrile seizures in P14 rat offspring [5]. These findings illustrate that the effects of prenatal stress on seizure susceptibility can vary depending on the type of stressor and the neurodevelopmental stage of the offspring. However, most studies have been conducted in infant and juvenile rats, but not in neonates, highlighting the need for further research to better understand the consequences of prenatal stress during the neonatal stage.

Given this background, our study aimed to evaluate the impact of maternal restraint stress during pregnancy on neonatal SE in rats. We assessed somatometric parameters, corticosterone blood levels, seizure severity, and neurodegeneration in neonatal P7 rat pups. We hypothesized that stress during central nervous system development would increase the sensitivity of the neonatal brain to seizures.

## 2. Materials and Methods

### 2.1. Study Subjects

All experiments were conducted in accordance with the ARRIVE guidelines and Mexican regulations (NOM-062-ZOO-1999) on the care and use of laboratory animals. The research received approval from the Internal Committee for the Care and Use of Laboratory Animals at the Instituto de Investigaciones Cerebrales (CICUAL-IICE 2021-003).

Wistar rats bred at the Instituto de Investigaciones Cerebrales of the Universidad Veracruzana were used for this project. The rats were maintained under controlled environmental conditions with a temperature of 23–25 °C and relative humidity of 65–75%. They were kept on a 12 h light-dark cycle (lights on at 08:00 h) and had ad libitum access to food and water. For mating purposes, adult, sexually experienced male rats were paired with nulliparous females that had no prior sexual experience. The estrous cycle of the females was determined through vaginal cytology. Mating involved placing a male and a receptive female in proestrus or estrus together for 24 h. If sperm was detected in the vaginal smears the following day, this was designated as gestational day (G) zero. Pregnant rats were housed with other females (four rats per cage) until G12. At that point, a group of rats underwent a restraint stress protocol. The pregnant rats continued to live with others from the same experimental group until gestational day 20, at which time they were moved to individual cages. The day of birth for the pups was marked as P0, and the number of pups per litter, as well as their sex, were recorded on this day. On P3, the litter size was adjusted to 10 pups (5 females and 5 males). The litters were kept with their mothers until the day following the induction of SE or the corresponding control manipulation, according to the experimental group.

### 2.2. Movement Restraint Stress

Stress was induced in pregnant rats from G12 to G20 by individually placing them in a cylindrical acrylic tube measuring 23 cm in length and 6.5 cm in diameter. This placement occurred twice a day: once from 10:00 AM to 12:00 PM and again from 3:00 PM and 5:00 PM, all under the light phase. In contrast, the rats of the control group were individually placed in a clean box with wood shavings for the same duration as the stressed group and remained in the housing room. After these manipulations, all rats were returned to their housing area. The body weight of all pregnant rats was recorded throughout gestation, from day 1 to day 20.

### 2.3. Experimental Groups

The first stage of the study involved monitoring pregnant mothers. A total of 16 pregnant rats were utilized in this phase of the study, with 8 rats assigned to each experimental group. Rats were categorized into the following experimental groups:

1. Control pregnant rats (control): These rats were maintained under standard housing conditions throughout gestation.
2. Pregnant rats in a stressful environment (stress): This group consisted of rats subjected to stress conditions due to movement restriction during gestation from days 12 to 20.

The second stage of the study focused on evaluating the offspring from the following experimental groups:

1. Offspring of control pregnant rats (control): These offspring were born to mothers that were kept in standard housing conditions during gestation.
2. Offspring of stressed pregnant rats (stress): This group included offspring whose mothers experienced stress from movement restriction during gestation days 12 to 20.

For this stage of the study, 8 pregnant rats from the previous phase were selected, with 4 rats per experimental group, resulting in a total of 40 offspring (20 males and 20 females) per treatment. This sample size was estimated using GPower software v3.1.9.6, considering an effect size of 0.4 for the mixed-effects ANOVA, with a significance level of 0.05 and a statistical power of 0.8.

### 2.4. Litter Characterization and Somatometric Evaluation

The body weight of mother rats in both the control and stress groups was monitored from day 1 to day 20 of gestation. The duration of gestation and the number of pups per litter, categorized by sex, were recorded on the day of birth (n = 8 per group). Measurements of body length (from the tip of the nose to the base of the tail) and body weight were taken from P3 to P7 (n = 4 litters per group, considering the litter as the experimental unit).

### 2.5. Blood Sample Collection and Determination of Serum Corticosterone

Blood samples were collected from the lateral tail vein of all pregnant rats on G12 and G20 to determine serum corticosterone concentration (n = 5 per group). Prior to blood collection, the tail was thoroughly cleaned with 70% ethanol to eliminate any fecal or urine residues. To minimize stress during the sampling procedure, 5% topical lidocaine was applied to the puncture site, as stress hormones and pain sensitivity can influence corticosterone levels. A sterile and disposable 22G (0.7 mm) needle was inserted into the lateral vein at a 45° angle in a posterior–anterior direction. Blood was collected by allowing it to drip until at least 0.5 mL was obtained, which was then deposited into amber SST microtainer tubes containing polymer gel to facilitate serum separation from the clot and fibrin. After the clot formed, the tubes were centrifuged at 18,894× *g* for 5 min. The serum was then collected and stored at −20 °C until further analysis.

For blood sample collection from neonates, 7-day-old control (n = 13) and stressed (n = 17) pups from four different dams per group were anesthetized with sodium pentobarbital (120 mg/kg, i.p.). Once unconsciousness was confirmed by the absence of the flexor reflex, each rat was placed in a dorsal recumbent position, and an incision was made to expose the thoracic area. Blood was extracted from the heart using a 1 mL syringe and a 27G (13 mm) needle. At least 0.4 mL of blood was collected and deposited into amber SST microtainer tubes (BD), which were subsequently centrifuged at 18,894× *g* for 5 min. The resulting serum was frozen at −20 °C until sample processing.

The determination of corticosterone levels was conducted using an ELISA kit designed for mouse and rat corticosterone (ALPCO, Salem, NH, USA, 55-CORMS-E01). Quantitative analysis was performed according to the manufacturer’s instructions, and the corticosterone concentration was determined from a calibration curve.

### 2.6. Induction of Status Epilepticus

Seizure activity was induced in rats using the lithium–pilocarpine model, as described by Torolira et al. [14]. On P6, rats from both experimental groups (n = 4 litters per group, considering the litter as the experimental unit) were injected with 5 mEq/kg lithium chloride (LiCl, Sigma-Aldrich, Toluca, Mexico) via intraperitoneal (i.p.). The following day, 1 mg/kg i.p. methylscopolamine (Sigma-Aldrich, Toluca, Mexico) was administered, followed 30 min later by subcutaneous (s.c.) injection of pilocarpine hydrochloride (Sigma-Aldrich, Toluca, Mexico) at a dosage of 320 mg/kg. Seizure severity was assessed using a modified version of the behavioral scale described by Torolira et al. [14]. The scale includes the following stages: Stage 1: Motor hyperactivity with myoclonus of the head and/or forelimbs. Stage 2: Clonic seizures accompanied by loss of posture and rotational movements. Stage 3: Continuous clonic seizures with vocalizations. Latency to the first stage 1, 2, or 3 seizure, the frequency of stage 1, 2, and 3 seizures over a period of 3.5 h, and the average seizure duration were evaluated. Seizure behavior was recorded on video for offline analysis. All rats treated with lithium–pilocarpine experienced SE, and no mortality associated with seizures was observed.

### 2.7. Evaluation of Neurodegeneration

Transcardial Perfusion. A total of 12 male and 12 female rats per group were included for brain histology (3 males and 3 females from each of 4 litters, to minimize potential litter effects). Twenty-four hours after seizures, rats were anesthetized with an overdose of sodium pentobarbital (120 mg/kg, intraperitoneally). Then, rats were positioned in a dorsal recumbent posture, and an incision was made at the level of the xiphoid appendix to expose the heart. They were perfused with 0.9% sodium chloride (NaCl), followed by phosphate-buffered 4% formaldehyde (pH 7.4). The perfusion flow rate was maintained between 15 and 20 mL/min.

Brain Tissue Processing for Histology*.* After completing the perfusion process, the brains were kept at 4 °C for 24 h. Each brain was then extracted from the cranial cavity and post-fixed in 4% paraformaldehyde for 2 h. Following this, the tissue was dehydrated using different concentrations of ethanol (ranging from 70% to 100%). The brains were then pre-embedded in paraffin for 2 h and ultimately embedded in paraffin blocks using a Leica dispenser (Wetzlar, Germany, model EG 1120). For sectioning, the brains were cut with a Leica microtome (Wetzlar, Germany, model RM2125RT). Coronal sections that were 15 µm thick were obtained from the frontal cortex (bregma −1.20 mm) to the dorsal hippocampus (bregma −2.60 mm) [17]. A total of twenty-four slides were prepared, each containing six coronal sections spaced 285 μm apart. The sections were mounted on gelatin-coated slides and stored at room temperature until needed.

Fluoro-Jade C (F-JC) Staining*.* F-JC staining was used to identify cells undergoing neurodegeneration [18]. Brain tissue sections were deparaffinized, and the staining process followed the instructions provided by the Ready-to-Dilute Staining Kit for identifying degenerating neurons (Biosensis, Thebarton, Australia). After drying the sections at 40 °C, they were dehydrated using varying concentrations of ethanol, cleared with xylene, and finally mounted using a non-aqueous mounting medium (Permount, Fisher Brand, Fair Lawn, USA). Histological analysis was conducted by visualizing the F-JC-stained brain sections with an Olympus AX70 epifluorescence microscope (Nagano, Japan). Counting of F-JC positive cells was done manually by directly identifying them under the microscope. Experimenters performing the histological analyses were blinded to treatment. The brain regions examined included the frontal cortex (F; AP, 2.6 mm from the bregma), the primary motor cortex (M1; AP, 1.2 mm from the bregma), cingulate cortex (Cg; (AP, 1.2 mm from the bregma), somatosensory cortex (S1BF; AP, −1.8 mm from the bregma), dorsal hippocampus (comprising areas CA1, CA2, CA3, hilus [H], and dentate gyrus [DG]; AP, −1.8 mm from the bregma), hypothalamus (Hypo; AP, −1.8 mm from the bregma), lateral thalamus (LT; AP, −1.8 mm from the bregma), ventral thalamus (VT; AP, −1.8 mm from the bregma), piriform cortex (Pir; AP, −1.8 mm from the bregma), and lateral amygdala (La; AP, −1.8 mm from the bregma) [16]. Three brain sections were evaluated for each brain region, and an average value of F-JC positive cells was obtained. We used the semi-quantitative score proposed by Torolira and colleagues [14]: “0” indicated 0 positive cells in the field, “1” indicated 1–15 cells, “2” indicated 16–30 cells, “3” indicated 31–50 cells, “4” indicated 51–100 cells, “5” indicated 101–200 cells, and “6” indicated >200 cells.

### 2.8. Statistical Analysis

Data normality was assessed using the Shapiro–Wilk test, except for the analyses of neurodegeneration and seizure frequency, which were evaluated using nonparametric tests. When the data did not follow a normal distribution, nonparametric tests were used for subsequent analyses, as detailed below. Results for males and females were analyzed separately.

Given the hierarchical structure of our data (pups nested within litters), we used litter means for primary statistical comparisons of body weight, body size, and behavioral variables associated with SE, while individual pup data were presented to illustrate biological variability. For the corticosterone and neurodegeneration analyses, we specifically sampled pups from each of the four litters per group to minimize potential litter bias while maintaining representation across all litters.

Maternal body weight during gestation (from G1 to G20) and offspring weight and size (measured from postnatal days 3 to 7) in the control and stress groups were compared using a two-way repeated measures ANOVA with two factors: (1) Treatment (control versus stress) and (2) Time (gestational or postnatal days). Tukey’s or Sidák’s post hoc tests were utilized to identify differences attributable to individual factors or their interactions.

Differences between experimental groups in the number of offspring born, gestation length, maternal serum corticosterone levels (measured on gestational days 12 and 20), and offspring corticosterone levels at P7 were analyzed using a two-tailed unpaired Student’s *t*-test. Differences in corticosterone levels between litters were compared using a one-way ANOVA followed by Tukey’s post hoc tests.

The number of F-JC positive cells in the brain regions of interest was compared between control and stress groups using the Mann–Whitney U test. Behavioral variables associated with SE were analyzed using either a two-tailed unpaired Student’s *t*-test or Mann–Whitney U test. Given the multiple comparisons performed on the seizure and neurodegeneration analysis, the resulting individual *p* values were adjusted using the Bonferroni method to control for Type I error inflation.

A significance level of *p* < 0.05 was considered. All statistical analyses were conducted using GraphPad Prism version 10.5 (La Jolla, CA, USA).

## 3. Results

### 3.1. Characteristics of Litters and Somatometric Evaluation

The body weight of mothers from both the control and stress groups was monitored from day 1 to day 20 of gestation. A two-way repeated measures ANOVA revealed significant differences in the mothers’ body weight due to gestational time (F(19, 140) = 16; *p* < 0.0001) and treatment (F(1, 140) = 160; *p* < 0.0001), as well as an interaction between these factors (F(19, 140) = 7.9; *p* < 0.0001). Multiple comparison tests showed that stressed mothers had significantly lower body weight (11.2–14.3%) compared to control mothers from G13 to G20 (*p* < 0.0001).

The duration of gestation was also recorded. The results indicated no significant differences in this parameter between the control (21.5 ± 0.26 days) and stressed (21.25 ± 0.36 days) groups (t = 0.5517, df = 14; *p* = 0.5899).

The total number of offspring born and the number of offspring by sex were recorded. The results indicated that the litter size was similar in both control and stressed mothers. The total number of pups was comparable (12.6 ± 0.59 for controls and 11.5 ± 0.5 for stressed mothers) (t = 1.448, df = 4; *p* = 0.1701). The number of male pups born was the same in both groups (7 ± 0.26 for both groups) (t = 0, df = 14; *p* > 0.999), while the number of female pups was similar (5.6 ± 0.59 for controls and 4.6 ± 0.53 for stressed mothers) (t = 1.252, df = 14; *p* = 0.2312).

**Figure 1:**
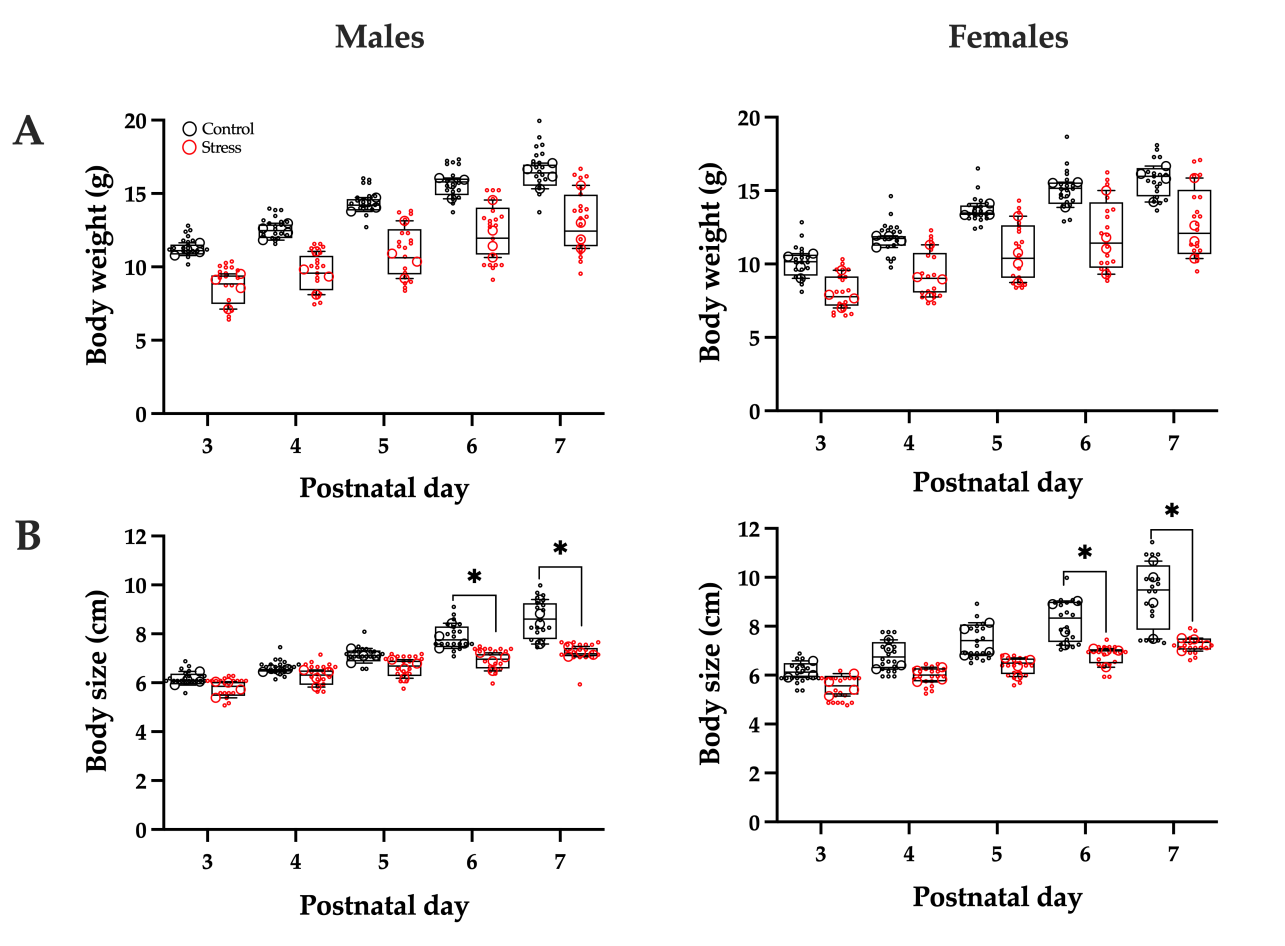
Body weight (**A**) and body size (**B**) of male and female rat pups from postnatal days 3 to 7, gestated under control or stress conditions. Data are from n=4 litters per group (20 pups per group: 10 males and 10 females). Individual data points represent either litter means (large circles) or individual pups (small circles), illustrating both within-litter and between-litter variability. Statistical analysis was performed using two-way repeated-measures ANOVA followed by Šidák’s post hoc test, considering the litter as the experimental unit. Males (left panels) and females (right panels) were analyzed separately. * *p* < 0.05 indicates a significant difference compared with the control group.

Pup body weight was monitored from P3 to P7. A two-way repeated measures ANOVA revealed significant main effects of age (F(1.369, 8.217) = 151.1; *p* < 0.0001) and treatment (F(1, 6) = 16.29; *p* < 0.0068) on male body weight, with no significant age × treatment interaction (F(1.369, 8.217) = 1.210; *p* = 0.3241). Similarly, in females, significant main effects were detected for age (F(4, 24) = 94.29, *p* < 0.0001) and treatment (F(1, 6) = 23.84, *p* < 0.0001), but not for the interaction between these factors (F(4, 24) = 1.24, *p* = 0.2565). Overall, both male and female pups from the stress group exhibited lower body weights than their respective controls (Figure 1A).

Body size was also assessed from P3 to P7. In males, a two-way repeated-measures ANOVA revealed significant main effects of age (F(4, 24) = 84.36, *p* < 0.0001) and treatment (F(1, 6) = 9.99, *p* = 0.0195), as well as a significant age × treatment interaction (F(4, 24) = 7.06, *p* = 0.0007). Post hoc analysis showed that males in the stress group were significantly shorter than controls at P6 (*p* = 0.0058) and P7 (*p* < 0.0001). In females, significant main effects of age (F(4, 24) = 62.54, *p* < 0.0001) and treatment (F(1, 6) = 8.27, *p* = 0.0282) were also observed, along with a significant age × treatment interaction (F(4, 24) = 5.30, *p* = 0.0033). Female pups in the stress group were shorter than controls at P6 (*p* = 0.0198) and P7 (*p* = 0.0005) (Figure 1B).

### 3.2. Serum Corticosterone Levels

**Figure 2:**
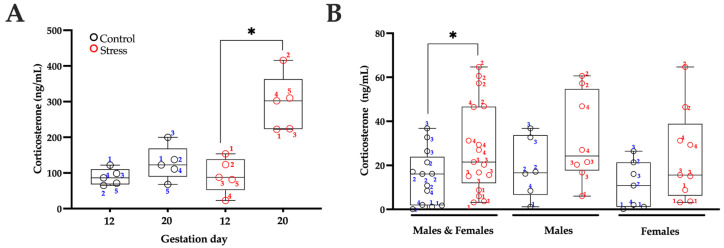
Serum corticosterone levels in pregnant mothers and offspring. Corticosterone blood levels in pregnant mothers maintained under standard conditions (control) and those subjected to restraint stress (stress) were assessed on gestational days 12 and 20 (**A**). Corticosterone levels in rat pups on postnatal day 7 were analyzed either combined for males and females or separately by sex; the same numbers were used to identify each offspring and its corresponding mother. (**B**). Data are represented as median and interquartile range, incorporating individual values. n = 5 mother per group; n = 13 control and 17 stressed pups from four different dams per group. A Student’s *t*-test was used for analysis, with * *p* < 0.05 indicating significance compared to the control group.

We analyzed corticosterone levels in mothers on G12 and G20. No significant differences were observed in the corticosterone concentrations of control mothers when comparing G12 and G20 (t = 1.904, df = 4; *p* = 0.1297). However, mothers subjected to movement restraint stress showed an increase in corticosterone levels on G20 (the end of the stress period) compared to G12 (prior to exposure to the stress protocol) (t = 4.68, df = 4; *p* = 0.0094; Figure 2A).

When examining serum corticosterone levels in the offspring, no significant differences were found between control and stressed male pups (t = 1.913, df = 12; *p* = 0.1913) and between control and stressed female pups (t = 1.534, df = 14; *p* = 0.1534). However, when combining the data from male and female rats in each treatment group, stressed rats exhibited higher corticosterone concentrations compared to control rats (t = 2.074, df = 28; *p* = 0.0474) (see Figure 2B).

Our results showed that pups born to stressed mothers with elevated corticosterone levels also exhibited higher concentrations of this hormone (Figure 2B). Moreover, significant differences in corticosterone concentrations were observed among control litters (F(3, 9) = 13.80, *p* = 0.0010) and among stressed litters (F(3, 13) = 33.60, *p* < 0.0001). Specifically, corticosterone levels in the offspring of control litter 1 were lower than those in litters 2 (*p* = 0.0329) and 3 (*p* = 0.0010), whereas litter 3 displayed higher levels than litter 4 (*p* = 0.0076). Among the stressed groups, pups from litter 1 had lower corticosterone concentrations than those from litters 2 (*p* < 0.0001) and 4 (*p* = 0.0018), while litter 2 exhibited higher levels than litters 3 (*p* < 0.0001) and 4 (*p* = 0.0015). These findings underscore the considerable variability in stress responsiveness among dams and, consequently, in the hormonal profiles of their offspring.

### 3.3. Seizure Severity

**Figure 3:**
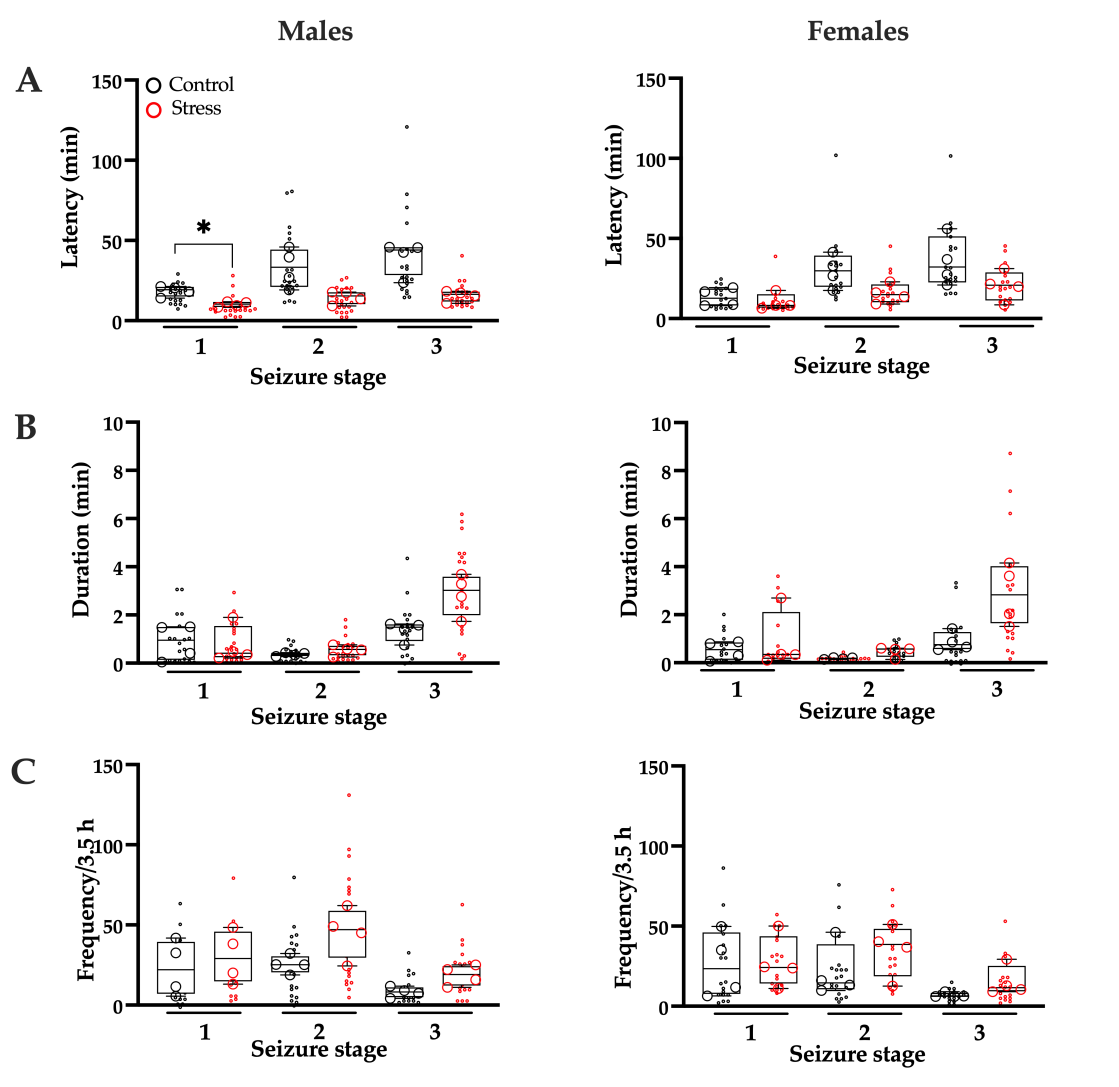
Seizure severity during *status epilepticus*. Latency (**A**), duration (**B**), and frequency (**C**) of seizures observed during *status epilepticus* in neonatal rats from the control and prenatally stressed (stress) groups. Data are from n = 4 litters per group (18–21 pups per group). Individual data points represent either litter means (large circles) or individual pups (small circles), illustrating both within-litter and between-litter variability. Statistical analysis was performed using Student’s *t*-test, with litter as the unit of analysis. Males (left panels) and females (right panels) were analyzed separately. * *p* < 0.0056 (Bonferroni correction) indicates significance compared with the control group.

Stressed males exhibited significantly shorter latencies for stage 1 seizures only, compared with control males. No differences were detected in overall seizure frequency or duration (Figure 3, Table 1). Stressed females showed similar stage 1, 2, and 3 seizure latency, frequency, and duration to control females (Figure 3, Table 2).

### 3.4. Neurodegeneration

**Figure 4:**
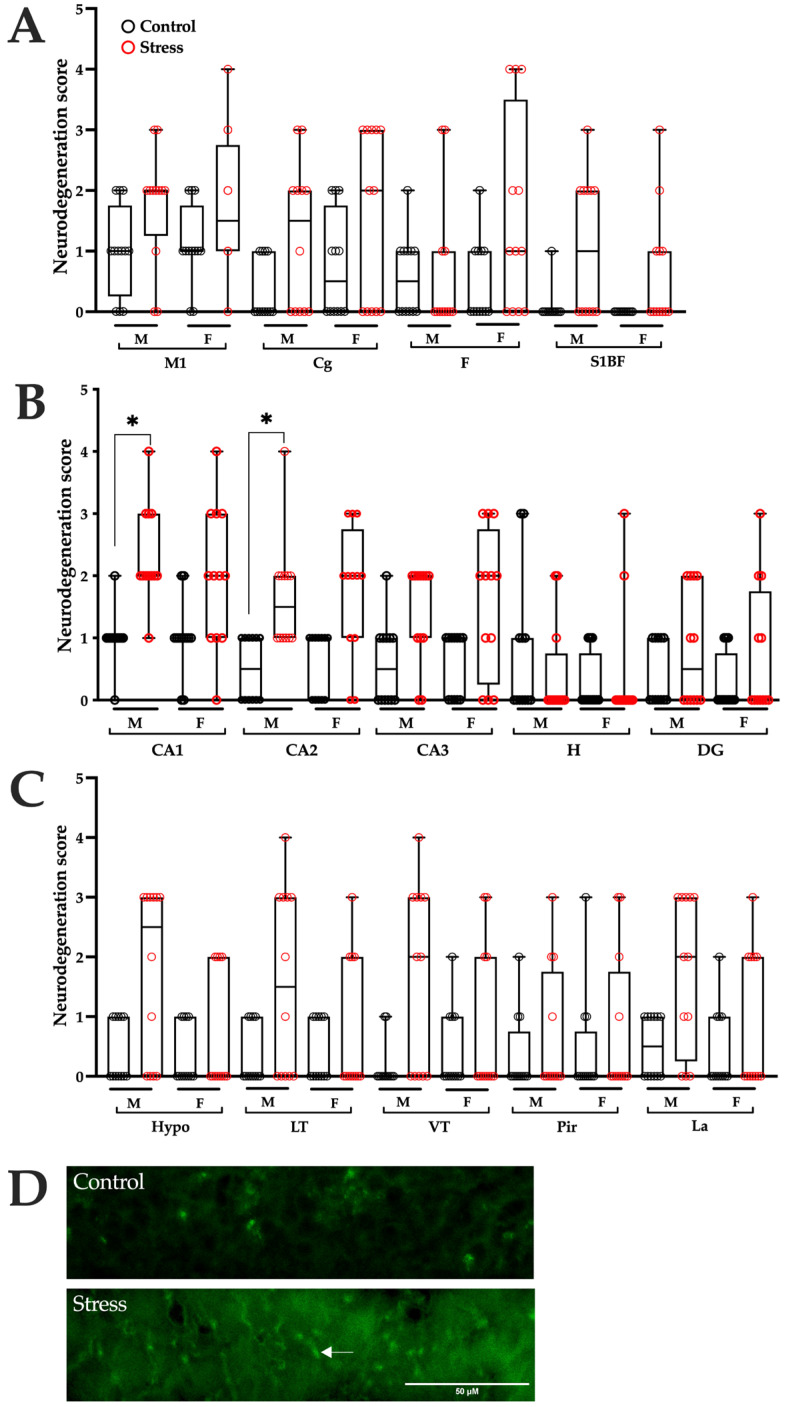
Neurodegeneration induced by status epilepticus (SE) in the brains of control and prenatally stressed rats. A semi-quantitative analysis, based on scores assigned according to the number of Fluoro-Jade C (F-JC) positive cells, identified injured cells in (**A**) primary motor cortex (M1), cingulate cortex (Cg), frontal cortex (F), and somatosensory cortex (S1BF); (**B**) dorsal hippocampus, including CA1, CA2, CA3, hilus (H), and dentate gyrus (DG); (**C**) hypothalamus (Hypo), lateral thalamus (LT), ventral thalamus (VT), piriform cortex (Pir), and lateral amygdala (La). (**D**) Representative photomicrographs (40×) of the CA1 region from control and stressed rats 24 h after SE. The arrow indicates an F-JC–positive cell. Scale bar = 50 µm. Data are presented as medians with interquartile ranges, and individual values are separated by sex (males: M, females: F). Statistical analysis was performed using the Mann–Whitney U test to compare experimental groups by sex. n = 12 male and 12 female rats per group (3 males and 3 females from each of 4 litters). * *p* < 0.0036 (Bonferroni correction) indicates significance when compared with the control group.

Analysis of F-JC-positive cells in brain tissue 24 h after SE revealed higher neurodegeneration scores in male rats gestated under stress conditions in the hippocampal CA1 and CA2 regions compared with control males. The remaining brain regions showed similar neurodegeneration scores in both experimental groups (Figure 4 and Table 1). No significant differences were observed between stressed and control females (Figure 4 and Table 2).

## 4. Discussion

Our findings indicate that pregnant rats exposed to restraint stress during the second and third weeks of gestation exhibited elevated corticosterone levels, which were also increased in their offspring. Prenatal stress exerted a moderate effect on seizure severity in the neonatal stage, with stressed male pups exhibiting more severe manifestations of SE and greater hippocampal neurodegeneration, whereas these adverse effects were not observed in females.

Pregnant rats experiencing movement restraint stress had lower weight gain compared to those maintained under standard conditions. Additionally, we observed that the stressed rat pups had reduced body weight and size compared to the control group. These findings align with observations made by Govindaraj and colleagues [19], who noted that pregnant rats exposed to immobilization stress experienced reduced weight gain during gestation, as well as lower birth weight and size in their offspring, along with a higher incidence of neonatal mortality. Similarly, Pardo et al. [20] reported detrimental effects induced by sleep restriction stress during pregnancy in rats.

In rat models of seizures, prenatal priming with betamethasone or exposure to acute immobilization stress has been shown to exacerbate seizure severity during the infant period [7,8,9,10,12]. This evidence partially aligns with our findings, as male neonatal rats exposed to prenatal stress exhibited reduced latency to stage 1 lithium–pilocarpine-induced seizures and extensive hippocampal neurodegeneration. In contrast, female rats did not display significant differences in seizure severity or neurodegeneration following SE.

Our study highlights the existence of sex-dependent differences in seizure susceptibility during early development, with males showing greater vulnerability to the adverse effects of gestational stress. The effects observed in males may reflect sex-specific interactions between prenatal stress, programming of the hypothalamic–pituitary–adrenal (HPA) axis, and the modulatory influence of sex hormones on neuronal excitability. Sadaghiani and Saboory (2010) reported that, although corticosterone levels were slightly higher in prenatally stressed female rats compared with males at P19, females exhibited greater resistance to pilocarpine-induced seizures [16]. Furthermore, accumulating evidence indicates that prenatally stressed males show more pronounced behavioral and neurobiological alterations than females, including impaired hippocampal neurogenesis and synaptic plasticity [21,22]. Males also exhibit higher seizure susceptibility in kainate, PTZ, and lithium–pilocarpine models, potentially due to the proconvulsant actions of testosterone and the anticonvulsant effects of estradiol and progesterone in females [23,24,25,26]. These mechanisms may underlie the sex bias observed in our results and underscore the need for future studies specifically powered to assess sex-by-treatment interactions.

The secretion of glucocorticoids represents a classic endocrine response to stress, influencing numerous physiological systems, including cardiovascular function, immunity, inflammation, metabolism, reproduction, and neurobiology [27]. Previous studies have shown that stress exposure during pregnancy in rats increases circulating levels of glucocorticoids, such as corticosterone and its metabolites, tetrahydrocorticosterone and 11-dehydrocorticosterone [5,15,20]. Consistent with these findings, our study supports a positive relationship between maternal and offspring corticosterone levels. Neonates born to mothers subjected to chronic physical space restriction throughout gestation exhibited higher serum corticosterone concentrations compared with offspring of control dams, in agreement with previous observations in infant rats [16].

Prenatal stress can disrupt fetal glucocorticoid homeostasis by reducing placental expression of 11β-hydroxysteroid dehydrogenase type 2 (11β-HSD2), thereby facilitating corticosterone transfer to the fetus [28,29]. Excessive fetal glucocorticoid exposure alters the developmental programming of the hypothalamic–pituitary–adrenal (HPA) axis, leading to hyperresponsiveness and impaired negative feedback regulation [30,31]. Elevated corticosterone levels during critical periods of brain maturation may also modify NKCC1/KCC2 expression and disrupt neuronal differentiation [10,32], generating a state of increased neuronal excitability. This mechanistic pathway may explain how prenatal corticosterone elevations contribute to HPA axis dysfunction and heightened seizure susceptibility early in life. Additional evidence suggests that the increased seizure severity observed following prenatal stress may arise from downregulation of genes encoding proteins critical for glutamatergic and GABAergic signaling within the arcuate nucleus of the medial and posterior hypothalamus [33]. Moreover, reductions in potassium–chloride cotransporter 2 (KCC2) and glutamic acid decarboxylase 67 expression have been reported in cortical tissue from P7 and P15 rats exposed to prenatal stress [9,10].

Excessive maternal glucocorticoid elevation during pregnancy can also alter glucocorticoid receptor expression in the neonatal hippocampus, a region that plays a central role in HPA axis regulation [34] and is particularly vulnerable to neuronal injury following SE [14]. Such alterations may contribute, at least in part, to the increased seizure susceptibility observed in our study. Notably, repression of hippocampal glucocorticoid receptors exacerbates hypoxic–ischemic injury in male, but not female, neonates, suggesting a potential sex-dependent role of glucocorticoids in neonatal brain vulnerability [35].

Our study has some limitations. The effects of prenatal stress on neonates were evaluated exclusively using the lithium–pilocarpine model, which predominantly engages the cholinergic system. While previous studies have shown that prenatal stress can also increase seizure susceptibility in hippocampal kindling and NMDA-induced spasm models [7,8,9,10,12], suggesting a broader involvement of glutamatergic and GABAergic pathways, the extent to which our findings generalize to other pro-epileptic mechanisms remains to be determined. Therefore, future studies employing PTZ- or kainate-induced seizures at age-appropriate doses will be essential to confirm whether the observed effects extend beyond the cholinergic pathway and to strengthen the translational breadth of this work. Potential sex-dependent effects also remain to be explored. Another limitation is the relatively small sample size (four litters per group), which, although scientifically appropriate to avoid pseudoreplication, may restrict statistical power. This constraint is inherent to developmental neuroscience studies, where the litter represents the true experimental unit and individual pups within the same litter cannot be considered independent observations. In addition, our findings reveal variability in maternal stress responsiveness and in the resulting hormonal profiles of the offspring. Although our sampling strategy and analyses were designed to minimize litter effects, we acknowledge that residual litter-specific influences, such as differences in maternal behavior [36] or intrauterine conditions [37,38], may contribute to the distinct patterns of seizure susceptibility and neurodegeneration observed across litters. Thus, while our results underscore the substantial impact of maternal stress, they also indicate that the litter itself may constitute an additional source of biological variability.

## 5. Conclusions

Overall, these results suggest that maternal restraint stress during pregnancy exerts only subtle, sex-dependent effects on neonatal vulnerability to seizures, primarily affecting males while leaving females unaffected. Although the magnitude of these effects was limited, they underscore the potential influence of early-life stress on the developing brain and highlight the need for further research into the mechanisms underlying this selective susceptibility.

## Figures and Tables

**Table 1:** Statistical parameters obtained from the analysis of seizures and neurodegeneration, comparing males in the stress group with those in the control group.

Variable	MWU/*t *Test	*p* Value	*p* Value Bonferroni Correction	Significant
Seizure behavior				
Latency to stage 1 seizure	*t* = 4.524, df = 6	0.004	0.0056	Yes
Latency to stage 2 seizure	*t* = 2.896, df = 6	0.0275		No
Latency to stage 3 seizure	U = 0	0.0286		No
Frequency of stage 1 seizure	U = 5	0.4857		No
Frequency of stage 2 seizure	U = 3	0.2		No
Frequency of stage 3 seizure	U = 1	0.0571		No
Duration of stage 1 seizure	*t* = 0.2386, df = 6	0.8193		No
Duration of stage 2 seizure	*t* = 1.582, df = 6	0.1646		No
Duration of stage 3 seizure	*t* = 3.269, df = 6	0.017		No
Neurodegeneration				
Frontal cortex (F)	U = 65	0.7179	0.0036	No
Primary motor cortex (M1)	U = 37.50	0.037		No
Cingulate cortex (Cg)	U = 42	0.0699		No
Somatosensory cortex (S1BF)	U = 39	0.0137		No
CA1	U = 9.500	<0.0001		Yes
CA2	U = 18	0.0007		Yes
CA3	U = 32	0.0244		No
Hilus (H)	U = 60	0.4638		No
Dentate gyrus (DG)	U = 56	0.2928		No
Hypothalamus (Hypo)	U = 36.5	0.0312		No
Ventral thalamus (VT)	U = 35	0.0116		No
Lateral thalamus (LT)	U = 42	0.0646		No
Piriform cortex (Pir)	U = 63	0.5505		No
Lateral amygdala (La)	U = 33	0.0236		No

**Table 2:** Statistical parameters obtained from the analysis of seizures and neurodegeneration, comparing females in the stress group with those in the control group.

Variable	MWU/*t* Test	*p* Value	*p* Value Bonferroni Correction	Significant
Seizure behavior				
Latency to stage 1 seizure	U = 5	0.4857	0.0056	No
Latency to stage 2 seizure	*t* = 2.428, df = 6	0.0513		No
Latency to stage 3 seizure	*t* = 1.684, df = 6	0.1432		No
Frequency of stage 1 seizure	U = 7	0.8857		No
Frequency of stage 2 seizure	U = 5	0.4857		No
Frequency of stage 3 seizure	U = 0.5	0.0571		No
Duration of stage 1 seizure	*t* = 0.5769, df = 6	0.585		No
Duration of stage 2 seizure	*t* = 2.596, df = 6	0.0409		No
Duration of stage 3 seizure	*t* = 2.980, df = 6	0.0246		No
Neurodegeneration				
Frontal cortex (F)	U = 44	0.1036	0.0036	No
Primary motor cortex (M1)	U = 52.5	0.2493		No
Cingulate cortex (Cg)	U = 48	0.1512		No
Somatosensory cortex (S1BF)	U = 42	0.0373		No
CA1	U = 33	0.0177		No
CA2	U = 26	0.0057		No
CA3	U = 35.5	0.0298		No
Hilus (H)	U = 69	>0.9999		No
Dentate gyrus (DG)	U = 55.5	0.2776		No
Hypothalamus (Hypo)	U = 64	0.6425		No
Ventral thalamus (VT)	U = 65	0.6700		No
Lateral thalamus (LT)	U = 68	0.9048		No
Piriform cortex (Pir)	U = 64	0.6044		No
Lateral amygdala (La)	U = 58	0.3871		No

## Data Availability

The raw data supporting the conclusions of this article will be made available by the authors upon request due to ethical and privacy considerations involving animal subjects.

## Abbreviations

The following abbreviations are used in this manuscript:

SE	*Status epilepticus*
P	Postnatal day
i.p.	Intraperitoneal
s.c.	Subcutaneous
F-JB	Fluoro-Jade B
F	Frontal cortex
M1	Primary motor cortex
Cg	Cingulate cortex
S1BF	Somatosensory cortex
CA1	CA1 hippocampal field
CA2	CA2 hippocampal field
CA3	CA3 hippocampal field
H	Hilus
DG	Dentate gyrus
Hypo	Hypothalamus
LT	Lateral thalamus
VT	Ventral thalamus
Pir	Piriform cortex
La	Lateral amygdala nucleus
ANOVA	Analysis of variance

## References

[1] Pascal R., Casas I., Genero M., Nakaki A., Youssef L., Larroya M., Benitez L., Gomez Y., Martinez-Aran A., Morilla I. (2023). Maternal Stress, Anxiety, Well-Being, and Sleep Quality in Pregnant Women throughout Gestation. J. Clin. Med..

[2] Karam F., Sheehy O., Huneau M.C., Chambers C., Fraser W.D., Johnson D., Kao K., Martin B., Riordan S.H., Roth M. (2016). Impact of maternal prenatal and parental postnatal stress on 1-year-old child development: Results from the OTIS antidepressants in pregnancy study. Arch. Womens Ment. Health.

[3] Scheinost D., Sinha R., Cross S.N., Kwon S.H., Sze G., Constable R.T., Ment L.R. (2017). Does prenatal stress alter the developing connectome?. Pediatr. Res..

[4] Crovetto F., Nakaki A., Arranz A., Borras R., Vellvé K., Paules C., Boutet M.L., Castro-Barquero S., Freitas T., Casas R. (2023). Effect of a Mediterranean Diet or Mindfulness-Based Stress Reduction During Pregnancy on Child Neurodevelopment: A Prespecified Analysis of the IMPACT BCN Randomized Clinical Trial. JAMA Netw. Open.

[5] Korgan A.C., Green A.D., Perrot T.S., Esser M.J. (2014). Limbic system activation is affected by prenatal predator exposure and postnatal environmental enrichment and further moderated by dam and sex. Behav. Brain Res..

[6] Fatima M., Srivastav S., Mondal A.C. (2017). Prenatal stress and depression associated neuronal development in neonates. Int. J. Dev. Neurosci..

[7] Chachua T., Yum M.S., Velíšková J., Velíšek L. (2011). Validation of the rat model of cryptogenic infantile spasms. Epilepsia.

[8] Yum M.S., Chachua T., Velíšková J., Velíšek L. (2012). Prenatal stress promotes development of spasms in infant rats. Epilepsia.

[9] Baek H., Yi M.H., Pandit S., Park J.B., Kwon H.H., Zhang E., Kim S., Shin N., Kim E., Lee Y.H. (2016). Altered expression of KCC2 in GABAergic interneuron contributes prenatal stress-induced epileptic spasms in infant rat. Neurochem. Int..

[10] Kwon H.H., Lee T., Hong J., Kim D.W., Kang J.W. (2018). Long-term prenatal stress increases susceptibility of N-methyl-D-aspartic acid-induced spasms in infant rats. Korean J. Pediatr..

[11] Bagheri M., Saboory E., Nejatbakhsh M., Roshan-Milani S., Derafshpour L., Sayyadi H., Rasmi Y. (2020). Prenatal stress increased γ2 GABAA receptor subunit gene expression in hippocampus and potentiated pentylenetetrazol-induced seizure in rats. Iran. J. Basic. Med. Sci..

[12] Edwards H.E., Dortok D., Tam J., Won D., Burnham W.M. (2002). Prenatal stress alters seizure thresholds and the development of kindled seizures in infant and adult rats. Horm. Behav..

[13] Sankar R., Shin D.H., Liu H., Mazarati A., Pereira de Vasconcelos A., Wasterlain C.G. (1998). Patterns of status epilepticus-induced neuronal injury during development and long-term consequences. J. Neurosci..

[14] Torolira D., Suchomelova L., Wasterlain C.G., Niquet J. (2016). Widespread neuronal injury in a model of cholinergic status epilepticus in postnatal day 7 rat pups. Epilepsy Res..

[15] Saboory E., Ahmadzadeh R., Roshan-Milani S. (2011). Prenatal exposure to restraint or predator stresses attenuates field excitatory postsynaptic potentials in infant rats. Int. J. Dev. Neurosci..

[16] Sadaghiani M.M., Saboory E. (2010). Prenatal stress potentiates pilocarpine-induced epileptic behaviors in infant rats both time and sex dependently. Epilepsy Behav..

[17] Khazipov R., Zaynutdinova D., Ogievetsky E., Valeeva G., Mitrukhina O., Manent J.B., Represa A. (2015). Atlas of the Postnatal Rat Brain in Stereotaxic Coordinates. Front. Neuroanat..

[18] Schmued L.C., Albertson C., Slikker W. (1997). Fluoro-Jade: A novel fluorochrome for the sensitive and reliable histochemical localization of neuronal degeneration. Brain Res..

[19] Govindaraj S., Shanmuganathan A., Rajan R. (2017). Maternal psychological stress-induced developmental disability, neonatal mortality and stillbirth in the offspring of Wistar albino rats. PLoS ONE.

[20] Pardo G.V., Goularte J.F., Hoefel A.L., de Castro A.L., Kucharski L.C., da Rosa Araujo A.S., Lucion A.B. (2016). Effects of sleep restriction during pregnancy on the mother and fetuses in rats. Physiol. Behav..

[21] Iturra-Mena A.M., Arriagada-Solimano M., Luttecke-Anders A., Dagnino-Subiabre A. (2018). Effects of prenatal stress on anxiety- and depressive-like behaviours are sex-specific in prepubertal rats. J. Neuroendocr..

[22] Soti M., Ranjbar H., Kohlmeier K.A., Shabani M. (2022). Sex differences in the vulnerability of the hippocampus to prenatal stress. Dev. Psychobiol..

[23] Mejías-Aponte C.A., Jiménez-Rivera C.A., Segarra A.C. (2002). Sex differences in models of temporal lobe epilepsy: Role of testosterone. Brain Res..

[24] Pollo M.L.M., Gimenes C., Covolan L. (2022). Male rats are more vulnerable to pentylenetetrazole-kindling model but females have more spatial memory-related deficits. Epilepsy Behav..

[25] Matovu D., Cavalheiro E.A. (2022). Differences in Evolution of Epileptic Seizures and Topographical Distribution of Tissue Damage in Selected Limbic Structures Between Male and Female Rats Submitted to the Pilocarpine Model. Front. Neurol..

[26] Wolf D.C., Desgent S., Sanon N.T., Chen J.-S., Elkaim L.M., Bosoi C.M., Awad P.N., Simard A., Salam M.T., Bilodeau G.-A. (2021). Sex differences in the developing brain impact stress-induced epileptogenicity following hyperthermia-induced seizures. Neurobiol. Dis..

[27] Sapolsky R.M., Romero L.M., Munck A.U. (2000). How do glucocorticoids influence stress responses? Integrating permissive, suppressive, stimulatory, and preparative actions. Endocr. Rev..

[28] Mairesse J., Lesage J., Breton C., Bréant B., Hahn T., Darnaudéry M., Dickson S.L., Seckl J., Blondeau B., Vieau D. (2007). Maternal stress alters endocrine function of the feto-placental unit in rats. Am. J. Physiol. Endocrinol. Metab..

[29] Welberg L.A., Thrivikraman K.V., Plotsky P.M. (2005). Chronic maternal stress inhibits the capacity to up-regulate placental 11beta-hydroxysteroid dehydrogenase type 2 activity. J. Endocrinol..

[30] Cottrell E.C., Seckl J.R. (2009). Prenatal stress, glucocorticoids and the programming of adult disease. Front. Behav. Neurosci..

[31] Jafari Z., Mehla J., Kolb B., Mohajerani M.H. (2017). Prenatal noise stress impairs HPA axis and cognitive performance in mice. Sci. Rep..

[32] Ehrlich D.E., Neigh G.N., Bourke C.H., Nemeth C.L., Hazra R., Ryan S.J., Rowson S., Jairam N., Sholar C.A., Rainnie D.G. (2015). Prenatal stress, regardless of concurrent escitalopram treatment, alters behavior and amygdala gene expression of adolescent female rats. Neuropharmacology.

[33] Iacobas D.A., Iacobas S., Chachua T., Goletiani C., Sidyelyeva G., Velíšková J., Velíšek L. (2013). Prenatal corticosteroids modify glutamatergic and GABAergic synapse genomic fabric: Insights from a novel animal model of infantile spasms. J. Neuroendocr..

[34] Jacobson L., Sapolsky R. (1991). The role of the hippocampus in feedback regulation of the hypothalamic-pituitary-adrenocortical axis. Endocr. Rev..

[35] Knox-Concepcion K.R., Figueroa J.D., Hartman R.E., Li Y., Zhang L. (2019). Repression of the Glucocorticoid Receptor Increases Hypoxic-Ischemic Brain Injury in the Male Neonatal Rat. Int. J. Mol. Sci..

[36] Lévy F., Keller M., Poindron P. (2004). Olfactory regulation of maternal behavior in mammals. Horm. Behav..

[37] Del Campo C.H., Ginther O.J. (1972). Vascular anatomy of the uterus and ovaries and the unilateral luteolytic effect of the uterus: Guinea pigs, rats, hamsters and rabbits. Am. J. Vet. Res..

[38] Liu G., Dong Y., Wang Z., Cao J., Chen Y. (2014). Restraint stress alters immune parameters and induces oxidative stress in the mouse uterus during embryo implantation. Stress.

